# Who Can Buffer Marginalization Risk? Affect Experience, Affect Valuation, and Social Marginalization in Japan and Brazil

**DOI:** 10.3389/fpsyg.2021.501165

**Published:** 2021-09-22

**Authors:** Igor De Almeida, Yukiko Uchida

**Affiliations:** ^1^Department of Human Coexistence, Graduate School of Human and Environmental Studies, Kyoto University, Kyoto, Japan; ^2^Kokoro Research Center, Kyoto University, Kyoto, Japan

**Keywords:** social marginalization, affect valuation, culture, East Asia, Latin America

## Abstract

Previous research has associated social marginalization with the rejection of mainstream cultural values. Since cultural values reflect affect valuation, the present research investigates the relationships between social marginalization and ideal/actual affect in two different non-WEIRD cultures, Brazil and Japan. As a social marginalization index, we used the NEET-Hikikomori Risk Scale (NHR). We predicted that cultural differences would emerge in the valuation of affective states. Affect valuation theory suggests that in East Asia, individuals are encouraged to pursue and value low arousal positive emotions (LAP: e.g., calmness, serenity) over high arousal positive emotions (HAP: e.g., excitement, elation, etc.) as they can harm social relationships in these societies. In contrast, Latin American cultures value HAP over LAP, because social relationships are promoted through vibrant positive emotional expression in these cultures. Hence, we hypothesized that individuals’ ideal affect, actual affect, and the discrepancy between ideal and actual affect would be associated with higher risk of social marginalization. Participants from Japan (*N* = 54) and Brazil (*N* = 54) reported their ideal affect and actual affect and completed the NEET-Hikikomori Risk Scale (NHR). Regression analyses showed that actual HAP and the discrepancy between ideal and actual HAP were negatively associated with NHR in Brazil, but no association was found in the Japanese data. The other variables, including ideal affect, were only minorly or not significantly associated with NHR. Though the study has limitations regarding its small sample size, we can explore future perspectives and discuss the relationships between emotion and cultural marginalization. Socioecological factors that promote actual HAP in Brazilians may encourage other mainstream cultural ideals, which buffers against cultural marginalization.

## Introduction

Since the 1990s, pressures stemming from globalization and long recessions have changed social and economic systems in most countries. In Japan, while business elites and senior government officials resisted pressures to change the labor market structure, a peripheral labor force, mostly composed of youth, has been pushed out of the system. Thus, Japanese youth compose the social stratum most prone to marginalization (see Toivonen et al., 2011 for a review).

Norasakkunkit and Uchida (2011, 2014) argue that marginalization can assume three forms – “freeter lifestyle orientation”, which refers to those who do not seek a full-time job, engaging in part-time jobs only; “NEET”, those “not in education, employment, or training”; and “*hikikomori*” (social withdrawal), a more extreme form of social marginalization, referring to those who do not take part in social interactions, even with family members, shutting themselves in their rooms for six months or longer.

Hikikomori is considered a more severe type of marginalization on the spectrum and was posited to be a culture-bound syndrome (Saito, 1998; Teo and Gaw, 2010), although similar cases have now been observed in countries outside of Japan. For instance, Kato et al. (2012) sent two *hikikomori* case vignettes to psychiatrists from Australia, Bangladesh, India, Iran, Japan, Korea, Taiwan, Thailand, and the United States. The psychiatrists were asked to rate the prevalence and etiology of the syndrome among other characteristics in their countries. Participants from all the studied countries perceived *hikikomori* as a present syndrome in their own countries, and most of them considered the *hikikomori* phenomenon to be associated with rapid socio-cultural changes derived from globalization. Other reports include cases in countries such as China (Wong et al., 2017), Spain (Ovejero et al., 2014), and Brazil (Gondim et al., 2017; Prioste and Siqueira, 2019). As such, the growing prevalence of such tendencies across the world requires additional investigation, especially in countries outside of Japan.

### Cultural Marginalization

Considering cultural marginalization as a spectrum encompassing freeters, NEET and *hikikomori*, Uchida and Norasakkunkit (2015) developed a scale to measure one’s tendency of becoming culturally marginalized, named the NEET-*Hikikomori* Spectrum Risk scale (NHR). Based on previous research, the authors identified psychological risk factors related to cultural marginalization and developed measurements for them. Their objective was to create an instrument that can measure the tendency to reject cultural values and become marginalized as a spectrum, so it allows researchers to analyze the mechanisms and processes of youth marginalization in society. In their studies, Uchida and Norasakkunkit (2015) compared individuals in three groups: those considered Hikikomori, those considered NEET, and workers. Those in the Hikikomori group scored the highest in NHR, followed by the NEET group and the workers group, all group differences being statistically significant. Despite these differences in score, no cut-off score for diagnosis was established in their article. This scale was used in several studies to further investigate the relationship between marginalization and rejection of cultural values. The scale consists of three subscales: “Freeter lifestyle preference”, “Lack of self-competence”, and “Unclear ambition for the future”. Freeter lifestyle preference refers to an inability to attain the cultural standard for becoming a socially sanctioned “adult” in society. “Lack of self-competence” encapsulates one’s confidence in social skills and academic and working skills. “Unclear ambition for the future” reflects whether individuals hold a clear vision of what they might want to do in the future. Norasakkunkit and Uchida (2011) showed that individuals scoring high on cultural marginalization tendency would score lower in interdependence on Singelis (1994)’s self-construal scale. Also, the same study showed that NHR was negatively associated with persistence after failure feedback, which is considered a mainstream Japanese behavior (see Heine et al., 2001), but not significantly positively associated with persistence after success feedback. This framework is similar to the theory of “cultural consonance”, which refers to the connection of an individual’s practices to shared cultural models, as proposed by Dressler (2007). An individual’s cultural consonance is assessed through the degree to which that individual, in their own beliefs and behaviors, approximates the socially shared expectations of their group.

As exemplified above, numerous studies relating NEET and hikikomori phenomena to an unwillingness to conform to mainstream cultural values during emerging adulthood have been done in Japan. However, studies on the same issue are rather less common in other cultures. Furthermore, while some researchers have investigated the relationship between mainstream values and social marginalization (e.g., Toivonen et al., 2011; Norasakkunkit and Uchida, 2014; Ishii and Uchida, 2016), to date no researcher has investigated how affective values are associated with social marginalization.

The above-mentioned literature suggests that a mismatch between mainstream culture and individuals’ own values might be associated with NEET and *hikikomori* psychological tendencies. Social marginalization occurs mostly in people during their late teenage years, twenties, or early thirties, a period of life conceptualized as emerging adulthood. This concept refers especially to young people in industrialized societies, who are between adolescence and adulthood, facing instability, exploring their own identity, self-focusing, feeling in-between, and contemplating possibilities (Arnett, 2007).

### Cultural Marginalization in the Brazilian Cultural Context

In Brazil, emerging adulthood, as a period of exploration of identity, the pursuit of diverse experiences, and the postponing of adult responsibilities, is particularly present in high socioeconomic status (SES) youth, who are more influenced by globalized values. Low SES youth, having limited access to the internet and tertiary education and thus being less influenced by globalized values, tend to comparatively assume traditional adult roles such as having a full-time job, focusing on their families, and having children and a stable partner (Dutra-Thomé and Koller, 2014).

Dressler et al. (2018) conducted a study in Brazil in which they measured individuals’ cultural consonance as well as the degree to which they knew about their society’s cultural model and compared these measures to their level of psychological distress. They found that both knowing about their society’s cultural model and having high levels of cultural consonance were associated with lower levels of psychological distress. However, cultural consonance acted as the stronger buffer for psychological distress.

The concept of NEET is getting particular attention in Brazil, where people in poverty, with fewer years of formal education, limited access to the internet, and a lower likelihood of being in a stable relationship have a higher probability of becoming NEETs due to the difficulties of securing a position in the current labor market without a high educational level, and lack of financial support to engage in other activities. Currently, the government has been implementing policies to try to mitigate this issue (Almeida and Figueiredo, 2017).

Cases of hikikomori are rather uncommon in Brazil, but there are some case studies. Gondim et al. (2017) described the case of a 25-year-old man, without previous history of psychiatric treatment; after breaking up with his girlfriend and quitting his job, the man stayed in his parents’ house for 29 years, having minimal contact with other people and rarely going outside. Prioste and Siqueira (2019) described the case of a 38-year-old man who had quit his job more than 10 years prior and refrained from social relationships for more than 5 years, spending his time playing computer games and browsing the internet. These cases showed some overlapping tendencies with cases in Japan.

### Affect and Culture

According to Affect Valuation Theory (Tsai et al., 2006; Tsai, 2007), affect can be divided into two types: “ideal affect”, which is what people idealize and would like to feel as shaped by mainstream cultural values, and “actual affect”, internal affective states that people actually feel in response to a situation. In general, culture guides which ideal affective states are pursued and valued more than others as they serve various functions, such as self-affirmation or relationship building, which are prioritized differently by different cultures. In turn, the consequences of the actual affect that individuals feel are determined by how in line they are with their culture’s ideal affect.

For example, the discrepancy between actual and ideal affect (i.e., the further away people are from culturally valued and desired affective states) is associated with greater intensity in depression (Tsai et al., 2006) and poorer psychological functioning (Tran et al., 2017).

In East Asian cultural contexts, individuals are encouraged to pursue and value low arousal positive emotions (LAP), such as being calm or serene. High arousal positive emotions (HAP), such as excitement and elation, are less desired since they can harm social relationships in these societies (Tsai et al., 2006). In Latin American cultural contexts, individuals follow an opposite pattern, valuing HAP over LAP, because social relationships are promoted through vibrant positive emotional expression in these societies (Ruby et al., 2012).

### Exploratory Study With Data From Brazil and Japan

As a small but important first step to see the difference between Brazilian and Japanese cultural marginalization and emotion, we conducted a survey study. Both cultures are commonly depicted in cultural psychology as collectivist (Triandis, 1995) and interdependence-fostering (Markus and Kitayama, 1991, 2010), in which the establishment and maintenance of social relationships are essential to the maintenance of order and development of social relationships in society, as well as the constitution of individuals’ selves. However, recently there has also been a growing body of research showing that East Asian and Latin American cultures have many differences when it comes to social and psychological features.

The function of emotions in each of these cultures as well as how they are valued varies (e.g., Ruby et al., 2012; De Almeida and Uchida, 2019). Therefore, we expected that each culture would display a specific relationship between social marginalization and affect.

This cultural difference is also reflected in cultural products such as song lyrics and news articles. A study comparing cultural products from Brazil and Japan showed that Brazilian products have more positive words, which can be related to HAP, than neutral and negative words, while Japanese products tend to have proportionally more neutral words, which can be related to LAP, than positive and negative words (De Almeida and Uchida, 2019). Hence, the current paper extends past literature by providing a new way of distinguishing two interdependent cultures, by comparing these cultures using ideal affect valuation.

We hypothesized that an individual’s affect (both ideal and actual) would be associated with the risk of being socially marginalized. Due to the cultural differences in affect valuation between the two cultures, we predicted that high actual and ideal HAP in Brazil would be connected to low marginalization risk in Brazil, while LAP would buffer against marginalization risk in Japan.

## Materials and Methods

### Participants

Due to the exploratory nature of this study, we opted for having university students as participants since previous studies suggested that there were more than 10% of university students at risk of marginalization, even including those in top elite universities in Japan (Norasakkunkit and Uchida, 2011) and in Singapore (Liew et al., 2021). University students in both Japan and Brazil are undergoing emerging adulthood, which is a time when people receive strong influence from globalization. In addition, since they are facing the pressure of getting a job after graduation, there is a risk of social marginalization. Thus university students can be studied as representatives of this particular life stage from each country. Also, despite this population being less prone to cultural marginalization, they are not immune to it; there are many reported cases of university students falling into social marginalization in several countries (e.g., Uchida, 2010; Bowker et al., 2019).

Participants were 54 Brazilian (30 females, mean age = 21.9, SD = 2.72) and 54 Japanese (21 females, mean age = 21.4, SD = 3.03) university students from various departments in top tier universities in each country (see Table 1). Brazilian participants were born and raised in Brazil and spoke Brazilian Portuguese as their first language, and Japanese participants were born and raised in Japan and spoke Japanese as their first language. Participants from neither group had lived for more than a year in a foreign country. Past or present psychiatric conditions were not asked about or measured.

**TABLE 1:** Participants demographic information.

Culture	N	Gender	Mage	SDage	SES
Brazil	54	30 F, 24 M	21.9	2.72	44 high SES, 10 low SES
Japan	54	21 F, 23 M	21.4	3.03	–

In the Japanese university, an announcement was posted on a bulletin board in one of the main buildings, as well as on an online version of the bulletin board. Participants came to the lab and answered the questionnaires on a laptop and received ¥500 Japanese yen (around 4 USD) for their participation.

Brazilian participants received invitations to participate through internal mailing lists. They answered the questionnaires outside the lab, and they did not receive compensation for their participation^1^. For this sample, information about the type of school the participants attended before university was also collected as a measure of SES. 44 participants attended only or mostly private schools (high SES), and 10 participants attended only public schools (low SES).

### Materials

Firstly, participants answered the Affect Valuation Index (AVI; Tsai et al., 2006). This index consists of two parts, both concerning 30 emotional states (enthusiastic, astonished, nervous, dull, quiet, relaxed, excited, surprised, elated, sleepy, still, lonely, strong, passive, content, sluggish, inactive, sad, euphoric, fearful, happy, idle, calm, unhappy, aroused, hostile, satisfied, rested, peaceful, and serene). In the first part, in order to measure ideal affect, participants answered how often they would ideally like to experience each emotional state over the course of a typical week on a 5-point scale (1-Never, 5-All the time). In the second part, participants rated how often they actually experienced each emotional state over the course of a typical week, using the same 5-point scale. For this study, we focused on high arousal positive affect (HAP – enthusiastic, excited, and strong) and low arousal positive affect (LAP – calm, at rest, relaxed, and serene), as classified by Tsai et al. (2006) in previous research.

Secondly, participants answered the NEET-*hikikomori* risk scale (NHR), developed by Uchida and Norasakkunkit (2015). It consists of 27 items (refer to the original article for details on each item) that participants rate on a 7-point scale (1- Completely disagree, 7- Completely agree), which are averaged to create the final score. This scale was made to assess NEET and hikikomori tendencies as a spectrum. It focuses on attitudes and values which suggest deviance from the cultural mainstream, commonly held by both groups.

Both materials already had a Japanese translation used in previous research. A Brazilian Portuguese version was made using back-translation.

Finally, participants provided some demographic information.

## Results

Statistical analyses of the present study were performed using the statistical programming language R.

**FIGURE 1:**
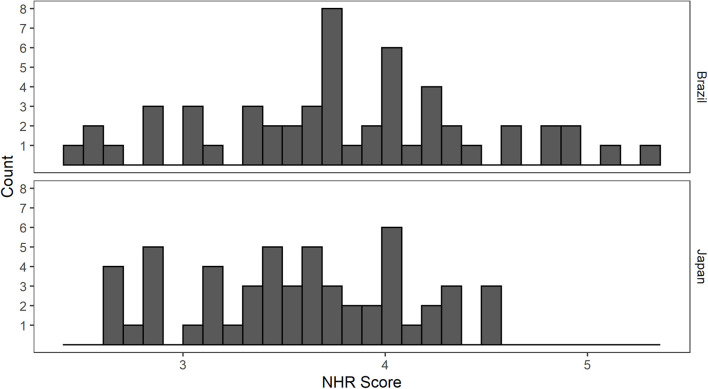
Histogram of NHR scores from each sample.

First of all, we examined the reliability of the NHR scale for Brazil, since it has only been validated for Japan and the United States. The Cronbach’s alpha score of the NHR was high for the Brazilian data (0.81), as it was for the Japanese data (0.78). There was no significant difference in NHR [*t*(106) = 1.95, *p* = 0.054] between the Brazilian group (*M* = 3.8, *SD* = 0.7) and the Japanese group (*M* = 3.6, *SD* = 0.5) (see Figure 1). Compared to the original study in which the scale was developed (Uchida and Norasakkunkit, 2015), both groups studied here scored similarly to the non-marginalized group (*M* = 3.5) and lower than the group classified as NEET (*M* = 4.4).

Next, to investigate cultural variability in ideal affect a 2 (Affect Type: HAP vs. LAP) × 2 (Culture: Brazil vs. Japan) mixed-model ANOVA with Affect Type as the within-subjects factor was conducted. There were significant effects for Culture, *F*(1,106) = 11.44, MSE = 0.43, *p* < 0.01, $\eta_{p}^{2}=0.06$, Affect Type, *F*(1,106) = 30.23, MSE = 0.29, *p* < 0.001, $\eta_{p}^{2}=0.10$, and the interaction between Culture and Affect Type, *F*(1,106) = 7.23, MSE = 0.29, *p* < 0.01, $\eta_{p}^{2}=0.02$. Bonferroni *p*-value adjusted *post hoc* tests were performed to evaluate Affect Type differences in each culture. In the Brazilian sample, LAP and HAP were not significantly different (*M* = 3.9 and *M* = 3.7, *p* = 0.1), however, Japanese participants scored significantly higher in LAP than HAP (*M* = 3.8 and *M* = 3.2, *p* < 0.001).

**FIGURE 2:**
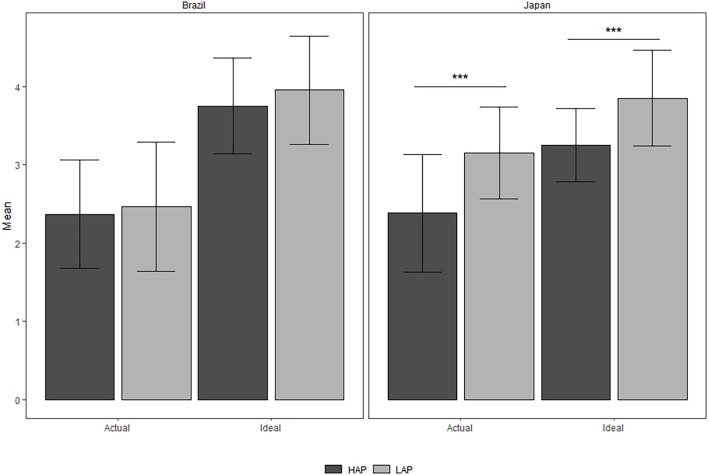
Comparison of actual and ideal HAP and LAP between Brazil and Japan (error bars represent standard deviation of the mean). ***p < 0.001.

The Japanese data replicated results from previous studies (Tsai et al., 2006), showing that Japanese, as has been commonly found for East Asians, would prefer LAP over HAP. However, the Brazilian data did not replicate previous findings considering Latin Americans (Ruby et al., 2012), since there was no significant difference in affective preference (see Figure 2).

Regarding actual affect, we conducted another 2 (Affect Type: HAP vs. LAP) × 2 (Culture: Brazil vs. Japan) mixed-model ANOVA with Affect Type as the within-subjects factor. There were significant effects for Culture, *F*(1,106) = 10.00, MSE = 0.65, *p* < 0.01, $\eta_{p}^{2}=0.05$, Affect Type, *F*(1,106) = 26.88, MSE = 0.38, *p* < 0.001, $\eta_{p}^{2}=0.08$, and the interaction between Culture and Affect Type, *F*(1,106) = 16.19, MSE = 0.38, *p* < 0.01, $\eta_{p}^{2}=0.05$. Bonferroni p-value adjusted *post hoc* tests were performed to evaluate Affect Type differences in each culture. In the Brazilian sample, LAP and HAP were not significantly different (*M* = 2.5 and *M* = 2.4, *p* = 0.37), however, Japanese participants scored significantly higher in LAP than HAP (*M* = 3.1 and *M* = 2.4, *p* < 0.001).

Finally, three multiple regression analyses were conducted to examine the effect of affect valuation and experience on social marginalization for each culture.

Each multiple regression followed the same general formula – NHR was predicted by HAP or LAP and their interaction with culture (Brazil or Japan). This way we could evaluate how HAP and LAP in ideal affect, actual affect, or the discrepancy between ideal and actual affect influences NHR in each culture, as well as the effect of culture on the model.

Firstly, the possible effects of ideal affect (high arousal positive and low arousal positive affect) on marginalization risk in each culture were evaluated through a regression model (culture was dummy coded). Contrary to our hypothesis, the results of the regression indicate that ideal HAP and ideal LAP did not explain the variance [*F*(5,102) = 1.5, *R*^2^ = 0.02, *p* = 0.2].

Secondly, the possible effects of actual affect (high arousal positive and low arousal positive affect) on marginalization risk in each culture were evaluated through a regression model (culture was dummy coded, see Table 2 for details). The model explained 23% of the variance [*F*(5,102) = 7.55, *R*^2^ = 0.27, *p* < 0.001,]. Culture did not have a significant effect, thus the two groups had similar scores on NHR. For the Brazilian sample, LAP (β = −0.20, *p* = 0.05) had a marginally significant influence and HAP (β = −0.40, *p* < 0.01) had a large influence. However, for the Japanese sample both LAP (β = −0.19, *p* = 0.71) and HAP (β = 0.25, *p* = 0.11) did not significantly influence NHR.

**TABLE 2:** Regression results using NHR as the criterion, actual affect as the dependent variable and culture (Brazil and Japan) as dummy variables.

Predictor	*b*	*b* 95% CI (LL, UL)	*sr* ^2^	*sr*^2^ 95% CI (LL, UL)	Fit
(Intercept)	5.24**	(4.66, 5.82)			
Culture	–0.50	(−1.60, 0.59)	0.01	(−0.02, 0.03)	
HAP (Brazil)	−0.40**	(−0.64, −0.16)	0.08	(−0.01, 0.16)	
LAP (Brazil)	–0.20	(−0.40, 0.00)	0.03	(−0.03, 0.08)	
HAP (Japan)	0.25	(−0.06, 0.57)	0.02	(−0.03, 0.06)	
LAP (Japan)	–0.06	(−0.38, 0.26)	0.00	(−0.01, 0.01)	
					*R*^2^ = 0.270**
					95% CI (0.10,0.37)

*A significant *b*-weight indicates the semi-partial correlation is also significant. *b* represents unstandardized regression weights. *sr*^2^ represents the semi-partial correlation squared. *LL* and *UL* indicate the lower and upper limits of a confidence interval, respectively.*

**p < 0.01.

Thirdly, the possible effects of the discrepancy between ideal and actual affect (raw values of ideal minus actual affect) on marginalization risk in each culture were evaluated through a regression model (culture was dummy coded). Results show that the regression model significantly explained the variance [*R*^2^ = 0.12, *p* < 0.01, *F*(5,102) = 3.96]. Similar to the actual affect model, culture did not have a significant effect. In the Brazilian sample, only the discrepancy in HAP (β = 0.40, *p* = 0.03) had a significant influence on NHR. In the Japanese sample, neither the discrepancy in HAP or LAP had a significant influence on NHR.

## Discussion

The study presented provides evidence that associates affect, as a mainstream cultural value, and the risk of social marginalization. This can be seen as a first step in this line of research, which can be fruitful not only for future studies, but also for the development of policies, and for professionals involved in applied work who design interventions and diagnose patients.

NEET-Hikikomori Risk Scale (NHR) had high reliability in this study’s Brazilian sample, showing that the latent variables are consistent and reliable in a culture outside of Japan. Future use of this scale in Brazilian culture in other facets of research may yield meaningful results. Future research can also expand the participant sampling pool to include those such as NEETs or people who are socially withdrawn to observe and analyze their psychological risk factors.

The present study adds to the literature with novel data from Brazil. We found that there is an association between the cultural valuation of affective states and social marginalization. The results of the regression models indicate that ideal HAP and ideal LAP did not explain marginalization risk in either culture. However, in the Brazilian sample (but not the Japanese sample), considering actual affect and the discrepancy between actual and ideal affect, low HAP predicted marginalization risk. LAP had a similar influence on marginalization risk, albeit only a marginally signiticant one. This implies that for Brazilians, actual positive affect has an important buffering effect toward marginalization risk. In particular, HAP is more likely to work as a buffer of marginalization risk in Brazil than in Japan. Actual affect and the discrepancy between ideal and actual affect had significant results, suggesting that it is the individual’s actual emotional state, not their ideal emotional state, that influences their cultural engagement. Considering that lived experiences are the major influence on one’s actual affect, one possible explanation for the present results is that environments and situations that promote actual HAP in Brazilians will also promote adherence to other mainstream cultural ideals, buffering against cultural marginalization. Future research may address this issue directly as well as exploring other consequences of one experiencing actual affect that goes against the ideal affect of mainstream culture.

Aside from our main hypothesis, this data suggests that for the mean score of ideal affect, Brazilian data (preferring LAP and HAP equally) ended up being similar to what was found in previous studies, in which, contrary to expectations, European-Americans (Tsai, 2007) and European-Canadians (Ruby et al., 2012) did not score significantly differently on ideal HAP and LAP. A recent study’s result has shown Colombians, European-Americans, and Japanese scoring significantly higher on ideal LAP than HAP, and Japanese scoring significantly lower than the other groups in HAP (Salvador et al., 2020). While questionnaire-based studies with participants sometimes show this pattern, behavioral and cultural products-based studies have consistently shown affective cultural values (e.g., Tsai et al., 2006; Tsai, 2007; De Almeida and Uchida, 2019). Therefore, there is the possibility that in the present study, the participants’ ideal affect was not a perfect reflection of what is expected in terms of affect in the Brazilian culture.

In addition, the previous studies mentioned here obtained data from Mexico (Ruby et al., 2012) and Colombia (Salvador et al., 2020), while we obtained data from Brazil. It is also arguable that valuing both HAP and LAP is a characteristic of Brazilian culture that differs from other Latin American cultures, or at least Colombia and Mexico. For instance, Zubieta et al. (1998) identified variables such as indigenous population, characteristics of the pre-Columbian cultures, socio-economic development, gender differences, and climate that can influence how emotions work in Latin American cultures. This difference between Latin American cultures further highlights the need for more studies comparing cultures within the same region rather than generalizing effects across cultural regions. Also, it suggests that the current usage of affective valuation as a functional means to explore cultural differences may have unique contributions compared to other more commonly used cultural measures.

Based on the divergence of the results from the present study and previous research on affect in Latin America, it is possible that the patterns and functions of emotions, as well as how and why they differ in each country, are simply not well understood. This could be due to a lack of studies focusing on this topic, especially when compared to the number of studies done with Western, or even East Asian populations.

Despite previous research suggesting that LAP supports social adjustment, while HAP may hinder social relationships in East Asian cultures (Tsai et al., 2006)—and LAP being considered a mainstream value in these societies and thus related to marginalization risk (Ishii and Uchida, 2016)—we could not find evidence that supports this hypothesis in our results. There are numerous possible explanations for this result; for instance, emotion could not be related to cultural marginalization in Japan, or this relationship could not be measurable in Japanese university students. Future research should investigate this further.

In summary, actual HAP can act as a buffer to cultural marginalization risk in Brazil, however, HAP and LAP may not be related to cultural marginalization risk in Japan. The current results should be interpreted in context, since they have limitations. First of all, the findings presented here may not be generalizable beyond the studied population, which consists of students from top tier universities (i.e., people with small chances of becoming socially marginalized) in each country, living in urban and economically developed regions of their countries, who are mostly high SES. Therefore, future studies must expand the findings of this study by addressing populations that are more at risk of becoming socially marginalized, or are at least not as privileged as the participants of this study. Secondly, other complementary measurements could be used for a better assessment of the situation, such as behavioral and physiological indices, psychiatric history, longitudinal measures, and cultural artifacts.

As globalization becomes more prevalent, societies around the world have to change accordingly, oftentimes pushing people into social marginalization. Despite being a global phenomenon, the present research shows that this process can happen in distinct ways according to local cultural values. Thus, future studies, as well as policies and perhaps treatments, can benefit from paying attention to how the relationship between cultural values and social marginalization develops in each society.

In a world where social marginalization is an important and frequently discussed topic (e.g., OECD, 2019), studies and policies tackling the issue from different angles are of urgent necessity. This study is a small but novel step in the direction of better understanding how this phenomenon works and, possibly, how it can be tackled in the future.

## Data Availability Statement

The datasets generated for this study are available on request to the corresponding author.

## Ethics Statement

The procedures used in this work were in accordance with the American Psychological Association Ethical Guidelines and the Japanese Psychological Association guidelines. All participants gave their informed consent and were debriefed and informed about the true purpose of the research immediately after the experiment.

## Author Contributions

Both authors contributed to the conception of the study and worked on the final version equally. ID did data collection, data analysis, and wrote the first draft. YU worked on the second version, contributed with interpretation of the data and theoretical review. Both authors contributed to the article and approved the submitted version.

## Conflict of Interest

The authors declare that the research was conducted in the absence of any commercial or financial relationships that could be construed as a potential conflict of interest.

## Publisher’s Note

All claims expressed in this article are solely those of the authors and do not necessarily represent those of their affiliated organizations, or those of the publisher, the editors and the reviewers. Any product that may be evaluated in this article, or claim that may be made by its manufacturer, is not guaranteed or endorsed by the publisher.

## Footnotes

1 It is a common practice in Brazilian universities to set up mailing lists within the university’s departments (e.g., “Graduate School of Engineering’s mailing list”), or students’ year (e.g., “class of 2012”). These mailing lists work as an alternative to bulletin boards as well as a direct means to deliver information to a whole class or department. They can be used for announcements and sharing information about things such as summer courses, internships, research grant opportunities, and recruitment for studies. It is customary to have voluntary participants in research in Brazil.

## References

[B1] Almeida J. B. S. A. D. Figueiredo A. M. R. (2017). POPULAÇÃO NEM-NEM: uma análise a partir dos dados da PNAD 2012[NEET POPULATION: an analysis from the National Household Sample Survey 2012]. *Rev. Estud. Soc.* 38 106–129. 10.19093/res4942

[B2] Arnett J. J. (2007). Emerging adulthood: what is it, and what is it good for? *Child Dev. Perspect.* 1 68–73.

[B3] Bowker J. C. Bowker M. H. Santo J. B. Ojo A. A. Etkin R. G. Raja R. (2019). Severe Social Withdrawal: cultural variation in past hikikomori experiences of University Students in Nigeria, Singapore, and the United States. *J. Genet. Psychol.* 180 217–230. 10.1080/00221325.2019.1633618 31305235

[B4] De Almeida I. Uchida Y. (2019). Examining affective valence in Japanese and Brazilian cultural products: an analysis on emotional words in song lyrics and newspapers. *Psychologia* 61 174–184. 10.2117/psysoc.2019-A103

[B5] Dressler W. W. (2007). “Cultural consonance,” in *Textbook of Cultural Psychiatry*, eds Bhugra D. Bhui K. (Cambridge: Cambridge University Press), 179–190.

[B6] Dressler W. W. Balieiro M. C. dos Santos J. E. (2018). What you know, what you do, and how you feel: cultural competence, cultural consonance, and psychological distress. *Front. Psychol.* 8:2355. 10.3389/fpsyg.2017.02355 29379460 PMC5775295

[B7] Dutra-Thomé L. Koller S. H. (2014). Emerging adulthood in Brazilians of differing socioeconomic status: transition to adulthood. *Paideia* 24 313–322. 10.1590/1982-43272459201405

[B8] Gondim F. A. A. Aragão A. P. Holanda Filha J. G. Messias E. L. M. (2017). Hikikomori in Brazil: 29 years of voluntary social withdrawal. *Asian J. Psychiatry* 30 163–164. 10.1016/j.ajp.2017.10.009 29065363

[B9] Heine S. J. Kitayama S. Lehman D. R. Takata T. Ide E. Leung C. (2001). Divergent consequences of success and failure in Japan and North America: an investigation of self-improving motivations and malleable selves. 81 599–615. 10.1037//0022-3514.81.4.599 11642348

[B10] Ishii K. Uchida Y. (2016). Japanese youth marginalization decreases interdependent orientation. *J. Cross Cult. Psychol.* 47 1–9. 10.1177/0022022115621969

[B11] Kato T. A. Tateno M. Shinfuku N. Fujisawa D. Teo A. R. Sartorius N. (2012). Does the “hikikomori” syndrome of social withdrawal exist outside Japan? A preliminary international investigation. *Soc. Psychiatry Psychiatr. Epidemiol.* 47 1061–1075. 10.1007/s00127-011-0411-7 21706238 PMC4909153

[B12] Liew K. Uchida Y. dela Cruz C. Lee L. N. (2021). Examining the cultural marginalisation theory of NEET/Hikikomori risk tendencies in Singaporean Youth. *Psychologia* 10.2117/psysoc.2020-A120

[B13] Markus H. R. Kitayama S. (1991). Culture and the self: implications for cognition, emotion, and motivation. *Psychol. Rev.* 98 224–253. 10.1037/0033-295X.98.2.224

[B14] Markus H. R. Kitayama S. (2010). Cultures and selves: a cycle of mutual constitution. *Perspect. Psychol. Sci.* 5 420–430. 10.1177/1745691610375557 26162188

[B15] Norasakkunkit V. Uchida Y. (2011). Psychological consequences of postindustrial anomie on self and motivation among Japanese youth. *J. Soc. Issues* 67 774–786. 10.1111/j.1540-4560.2011.01727.x

[B16] Norasakkunkit V. Uchida Y. (2014). To conform or to maintain self-consistency? Hikikomori risk in Japan and the deviation from seeking harmony. *J. Soc. Clin. Psychol.* 33 918–935. 10.1521/jscp.2014.33.10.918

[B17] OECD (2019). *Education at a Glance 2019: OECD Indicators.* Paris: OECD Publishing.

[B18] Ovejero S. Caro-Cañizares I. de León-Martínez V. Baca-Garcia E. (2014). Prolonged social withdrawal disorder: a hikikomori case in Spain. *Int. J. Soc. Psychiatry* 60 562–565. 10.1177/0020764013504560 24101742

[B19] Prioste C. D. Siqueira R. C. D. (2019). Fetichismo virtual na vida de um hikikomori brasileiro: um estudo de caso. *Doxa: Rev. Bras. Psicol. Educ.* 21 4–16. 10.30715/doxa.v21i1.12931

[B20] Ruby M. B. Falk C. F. Heine S. J. Villa C. Silberstein O. (2012). Not all collectivisms are equal: opposing preferences for ideal affect between East Asians and Mexicans. *Emotion* 12 1206–1209. 10.1037/a0029118 22775131

[B21] Saito T. (1998). *Shakaiteki Hikikomori: Owaranai Shishunki [Social Withdrawal: Unending Adolescence].* Tokyo: PHP Shuppan.

[B22] Salvador C. E. Carlier S. I. Ishii K. Castillo C. T. Nanakdewa K. Savani K. (2020). Expressive interdependence in Latin America: a Colombia, U.S., and Japan comparison. PsyArXiv [Preprint]. 10.31234/osf.io/pw4yk

[B23] Singelis T. M. (1994). The measurement of independent and interdependent self-construals. *Pers. Soc. Psychol. Bull.* 20 580–591. 10.1177/0146167294205014

[B24] Teo A. R. Gaw A. C. (2010). Hikikomori, a Japanese culture-bound syndrome of social withdrawal?: a proposal for DSM-5. *J. Nerv. Ment. Dis.* 198 444–449. 10.1097/NMD.0b013e3181e086b1 20531124 PMC4912003

[B25] Toivonen T. Norasakkunkit V. Uchida Y. (2011). Unable to conform, unwilling to rebel? Youth, culture, and motivation in globalizing Japan. *Front. Psychol.* 2:207. 10.3389/fpsyg.2011.00207 21949510 PMC3171786

[B26] Tran A. G. T. T. Su J. C. Chong S. S. Woei-Haur L. (2017). When the grass could be greener: psychological correlates of positive affect discrepancies in white american and taiwanese samples. *J. Cross Cult. Psychol.* 48 931–949. 10.1177/0022022117703484

[B27] Triandis H. C. (1995). *Individualism and Collectivism.* Boulder, CO: Westview Press.

[B28] Tsai J. L. (2007). Ideal affect: cultural causes and behavioral consequences. *Perspect. Psychol. Sci.* 2 242–259. 10.1111/j.1745-6916.2007.00043.x 26151968

[B29] Tsai J. L. Knutson B. Fung H. H. (2006). Cultural variation in affect valuation. *J. Pers. Soc. Psychol.* 90 288–307. 10.1037/0022-3514.90.2.288 16536652

[B30] Uchida C. (2010). Apathetic and withdrawing students in Japanese Universities -with regard to Hikikomori and Student Apathy -. *J. Med. Dent. Sci.* 57 95–108. 10.11480/jmds.570111 20437770

[B31] Uchida Y. Norasakkunkit V. (2015). The NEET and Hikikomori spectrum: assessing the risks and consequences of becoming culturally marginalized. *Front. Psychol.* 6:1117. 10.3389/fpsyg.2015.01117 26347667 PMC4540084

[B32] Wong P. W. C. Liu L. L. Li T. M. H. Kato T. A. Teo A. R. (2017). Does hikikomori (severe social withdrawal) exist among young people in urban areas of China? *Asian J. Psychiatry* 30 175–176. 10.1016/j.ajp.2017.10.026 29080513

[B33] Zubieta E. Fernández I. Vergara A. I. Martínez M. D. Candia L. (1998). Cultura y emoción en Amŕrica [Culture and emotion in the Americas]. *Boletín de Psicología*. 61 65–90.

